# Implementing Artificial Intelligence in Radiology: Design Thinking Road Map

**DOI:** 10.2196/87360

**Published:** 2026-04-29

**Authors:** Vitor Ulisses Monnaka, Jéssica Andrade-Silva, Gilberto Szarf, Henrique Min Ho Lee

**Affiliations:** 1 Department of Innovation, Hospital Israelita Albert Einstein, Avenida Albert Einstein, 627/701, São Paulo, 05652-900, Brazil, 55 1121511233; 2 Department of Radiology, Hospital Israelita Albert Einstein, São Paulo, Brazil

**Keywords:** artificial intelligence, radiology, design thinking, clinical implementation, human-centered design, technology adoption

## Abstract

Despite its promising potential to transform medical care, particularly in the field of medical images, the integration of artificial intelligence (AI) into clinical practice remains a complex and multifaceted challenge. In real-world settings, AI tools may demonstrate limited clinical impact, suboptimal performance, and security vulnerabilities, and face regulatory constraints. This viewpoint explores how the principles of design thinking can provide a structured road map for AI implementation in radiology. By emphasizing user-centeredness, fostering multidisciplinary collaboration, and embedding iterative refinement, this approach offers practical guidance for identifying clinical and operational needs, selecting and validating appropriate solutions, and ensuring effective deployment with continuous improvement.

## Introduction

The adoption of artificial intelligence (AI) holds great promise for improving several aspects of medical care [1]. Radiology stands out as the leading specialty in AI and machine learning patents, with 1160 patents filed worldwide over the past decades [2]. These solutions span a wide range of functions, from automation of repetitive tasks and image interpretation to workflow orchestration [3], contributing to a market projected to reach US $21 billion by 2030 [4].

Despite this promising landscape, integrating AI tools into clinical practice remains highly challenging [5]. Performance observed in real-world practice is often lower than the metrics reported in algorithm development settings [6]. Additional barriers include concerns about data privacy and regulatory compliance [7], lack of interoperability with existing institutional workflows [8], and insufficient user engagement [9]. Previous studies have appropriately highlighted the importance of governance structures and technical pipelines for AI implementation [5,10]. However, perhaps most critically, even AI solutions that meet all technical and regulatory requirements may fail to deliver meaningful impact if they are not aligned with the specific needs of the institution where they are deployed [11].

This gap between innovation and impact is a common dilemma in the implementation of new technologies: tools may be technically sophisticated but fail to generate value if they do not solve real-world user problems. Just as not all problems have solutions, not all solutions solve real problems. To address this challenge, we proposed using design thinking as a guiding road map for implementing AI in radiology [12].

**Figure 1.**
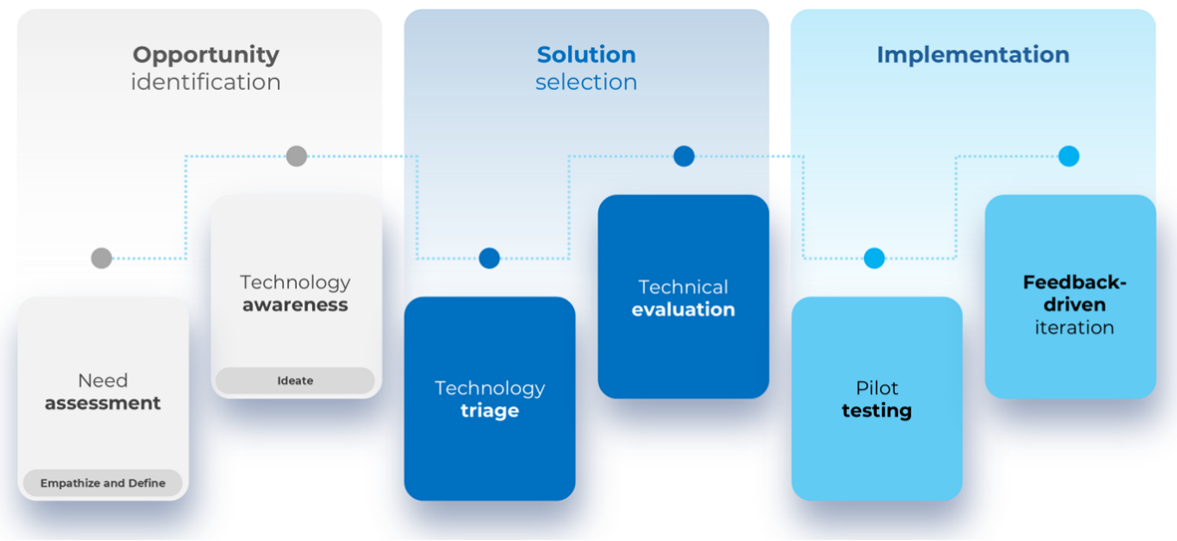
Design thinking–informed road map for artificial intelligence adoption in radiology. The process progresses from opportunity identification (need assessment and technology awareness) to solution selection (technology triage and technical evaluation) and implementation (pilot testing and feedback-driven iteration).

Although originally developed in the fields of product and service design, this structured and iterative approach has been widely adopted to solve complex problems through human-centered strategies, collaborative decision-making, and continuous refinement [13]. By closely examining stakeholder experiences, specific sources of dissatisfaction or frustration can be identified, guiding the selection of solutions that offer meaningful impact and a greater likelihood of adoption.

Importantly, existing AI implementation frameworks offer limited practical guidance on how to operationalize user-centered principles throughout the decision-making process. This gap has contributed to the frequent adoption of AI solutions that demonstrate technical promise but fail to generate measurable clinical or operational value, leading to inefficient use of institutional resources [14]. To address this limitation, this viewpoint provides a practical operationalization of design thinking principles across concrete phases (opportunity identification, solution selection, and implementation) for AI implementation in radiology (Figure 1).

## Opportunity Identification

### Need Assessment

**Figure 2.**
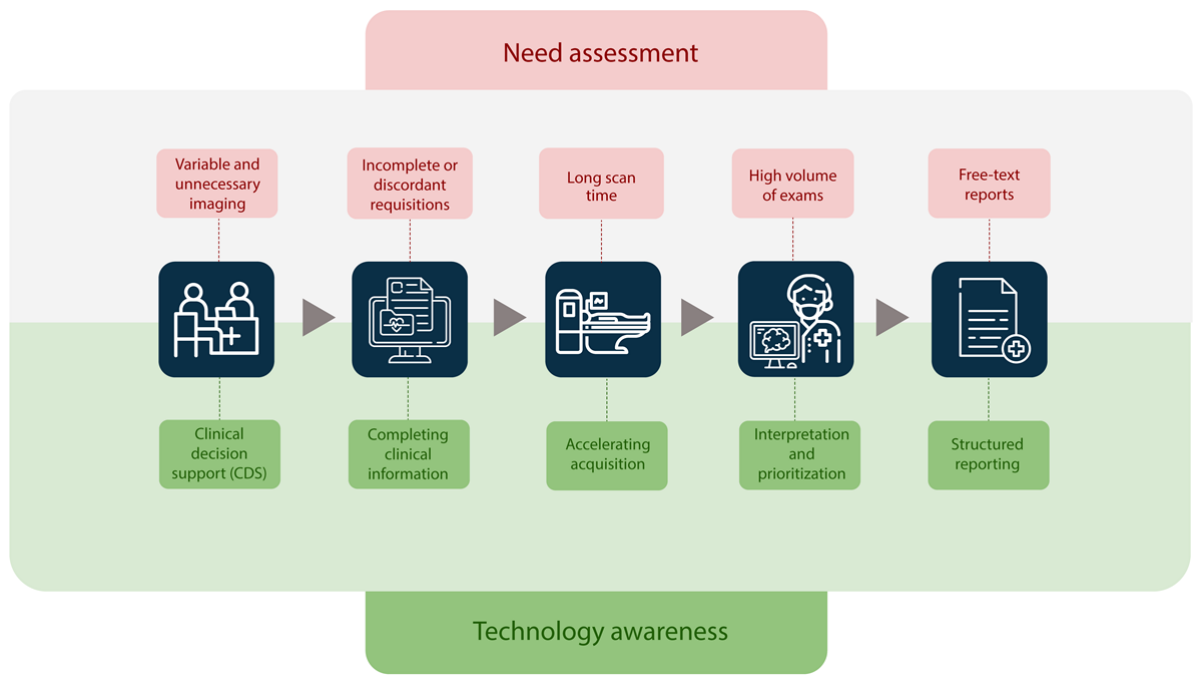
Identification of opportunities for artificial intelligence (AI) implementation in radiology. The need assessment phase involves an in-depth analysis of institutional workflows to identify pain points (some of them exemplified in red) experienced by stakeholders. Awareness of AI capabilities (in green) can reveal latent opportunities by reframing performance expectations and guiding the prioritization of impactful solutions.

The design thinking framework begins with an empathetic, immersive exploration of the real-world challenges faced by users. In radiology, this phase starts with a comprehensive assessment of institutional workflows to understand the people, processes, and systems involved, including the types of exams performed, clinical specialties covered, and reporting routines (Figure 2). Building on such mapping, structured interviews and co-design workshops with radiologists and other end users help to identify the most significant pain points in each stage of the workflow. The structuring and prioritization of these observed challenges correspond to the define phase of design thinking. A previous study detailed a structured approach to workflow mapping in radiology, identifying operational inefficiencies that were then prioritized according to their frequency and impact on clinical, financial, and staff well-being outcomes [15].

At our institution, the process of need assessment is conducted jointly by the radiology and innovation departments (Table 1). Following workflow mapping, we organize open discussion forums with clinical staff and end users, with each session focused on a specific stage of the workflow. This broad participation fosters a sense of ownership over future solutions, reducing resistance to adoption and promoting long-term engagement. Recurring pain points identified in these forums include work overload, radiologist fatigue, and repetitive, time-consuming activities such as clerical tasks and manual measurements.

It is also important to recognize that problems and needs are socially and technologically constructed. A situation is often only framed as a “problem” once an improved alternative is perceived as feasible. Without this perceived gap between what is and what could be, long-standing inefficiencies tend to be normalized as immutable aspects of clinical practice. Conversely, new innovations can retroactively cast previously accepted practices as suboptimal. For instance, the introduction of AI-based automated worklist prioritization has shifted perceptions of manual triage, which was once considered adequate.

Thus, while problem-driven approaches are essential to ensure user-centered and clinically relevant AI adoption, technology awareness plays a pivotal role in reshaping tolerance for existing inefficiencies. We, therefore, recommend that open forums not only validate user-reported pain points but also include discussions of existing technological solutions, assessing whether they can effectively address the specific needs of the institution.

In this sense, although traditional design thinking frameworks transition from problem definition to ideation as a stage for generating novel solutions, in the context of health care technology implementation, this phase often takes the form of structured technology scouting. Thus, rather than developing entirely new solutions, institutions systematically explore available and emerging tools aligned with identified needs.

**Table 1.** Key phases, activities, and stakeholders involved in the implementation of artificial intelligence (AI) in radiology.

Phase	Need assessment	Technology selection	Technical evaluaton
Activities	Mapping institutional workflows and validating pain points	Selection of commercially available AI solutions	Assessing solution architecture, data protection, and cybersecurity
Stakeholders	Radiology and innovation departments	Governance structure (radiologists, data scientists, IT professionals, and hospital administrators)	Multidisciplinary IT team (expertise in systems architecture, data governance, privacy compliance, and information security)

### AI Use Cases in Radiology

Given the importance of technology awareness in uncovering latent opportunities for improvement, this section outlines some of the main use cases of AI in radiology (Figure 1, in green). Although image interpretation has received the most emphasis, growing evidence highlights AI’s potential across the entire imaging process [10].

At the preimaging stage, clinical decision support (CDS) algorithms are assisting physicians in exam ordering by considering the patient’s specific clinical scenario, including determining whether imaging is indicated and identifying the most cost-effective modality [16]. These tools aim to optimize resource use, decrease practice variability, and improve adherence to protocols. A commercial CDS platform integrated with electronic medical records (EMRs) [17] reduced the frequency of low-utility imaging studies (from 11% to 5.4%) and increased the proportion of appropriate studies (from 64.5% to 82%). Although still under development, deep learning (DL) techniques aim to leverage large-scale data from EMRs by integrating diverse clinical inputs to generate patient-specific recommendations [18,19].

Another promising preimaging application is the use of large language models to automatically generate structured clinical histories from EMR notes to accompany imaging requisitions, which frequently lack essential clinical information, with up to 81% found to be incomplete and 42% discordant when compared to corresponding provider notes in the EMR [20]. These discrepancies can negatively impact exam planning and, more importantly, the accuracy of radiologic interpretation. In a recent study [21] involving oncologic computed tomography (CT) exams, GPT-4 significantly outperformed physician-written histories in completeness, being preferred by radiologists in 89% of cases.

In image acquisition, DL reconstruction techniques significantly reduce magnetic resonance imaging (MRI) acquisition times without compromising image quality [22]. Among clinical applications, musculoskeletal radiology has been the most impacted, given its demand for high spatial resolution and precise tissue characterization [23]. One study showed that DL-based reconstruction enabled a comprehensive 5-minute knee MRI at 3 T, with image quality interchangeable with standard images and diagnostic agreement preserved over 96% of the time for any feature evaluated [24]. Similarly, another prospective validation of a DL-accelerated knee MRI protocol demonstrated that scan time could be nearly halved compared to standard imaging while maintaining image quality and diagnostic equivalence [25].

In the domain of image interpretation, AI tools are increasingly used to enhance diagnostic accuracy and prioritize urgent cases. DL algorithms have demonstrated efficacy in detecting fractures on radiographs, often achieving performance comparable to that of emergency physicians and radiologists [26,27]. Similarly, AI assistance has improved the detection of thoracic abnormalities on chest radiographs, particularly in high-volume clinical settings [28]. In parallel, AI tools have proven valuable for triaging and prioritizing critical findings. For instance, deep neural networks have been used to flag abnormal chest radiographs for expedited review [29]. Furthermore, a prospective study demonstrated that an AI system for CT pulmonary angiography identified pulmonary emboli, decreasing waiting times for positive studies [30]. These studies underscore the growing role of AI as an assistive tool to support radiologists in managing increasing imaging volumes.

AI is also driving significant advancements in radiology reporting, with natural language processing tools enabling the extraction of structured data from free-text reports. Structured reporting improves communication, fosters collaboration among medical professionals, and standardizes reporting language between institutions [31]. A recent study demonstrated the potential of GPT-4 in autonomously selecting the most appropriate structuring template before extracting findings, successfully converting all tested free-text reports into valid JSON files without errors [32]. Another study integrated natural language processing with speech recognition to develop a reporting tool capable of real-time conversions of dictation into structured reports, achieving high accuracy in the context of urolithiasis CT reporting [33]. Overall, these innovations enhance report clarity, reduce interobserver variability, and enable more actionable and interoperable documentation across health care systems.

## Solution Selection

### Governance Structure

Resource limitations impose the necessity of defining which opportunities should be prioritized, a process facilitated by a governance structure: a formalized system of roles and procedures that guides a multidisciplinary team in collaborative decision-making for technology selection (Table 1).

Governance structures should ideally comprise a multidisciplinary coalition of stakeholders, including radiologists, data scientists, IT professionals, and hospital administrators. This diversity of expertise is essential to balance clinical imperatives with operational constraints and regulatory requirements. Radiologists offer in-depth knowledge and insight into workflow demands and clinical priorities; data scientists bring expertise in algorithm development, model validation, and performance monitoring; IT professionals oversee systems integration, cybersecurity, and infrastructure readiness; and administrators ensure alignment with institutional strategies, regulatory compliance, and resource allocation.

The assembly of a multidisciplinary governance team faces several barriers. In practice, fragmentation between departments, competing priorities, and siloed decision-making structures often lead to duplicated efforts and suboptimal allocation of resources [34-36]. Conversely, institutions that embed AI initiatives within a unified organizational strategy demonstrate better coordination across administrative units and more effective distribution of funding [5]. Accordingly, active involvement of executive leadership is critical to incorporate AI initiatives into the broader strategic agenda of the health care organization and to legitimate institutional commitment [37]. Leadership sponsorship can help overcome organizational silos by promoting formal cross-department governance committees, standardized communication channels, and shared key performance indicators (KPIs) across departments [35]. Importantly, this institutional commitment must be reflected in tangible investments, from infrastructure planning to allocation of dedicated resources, including protected time for clinicians to participate in governance activities and sustained investment in a devoted workforce, particularly in key roles such as data scientists and informaticians.

### Technology Triage

**Figure 3.**
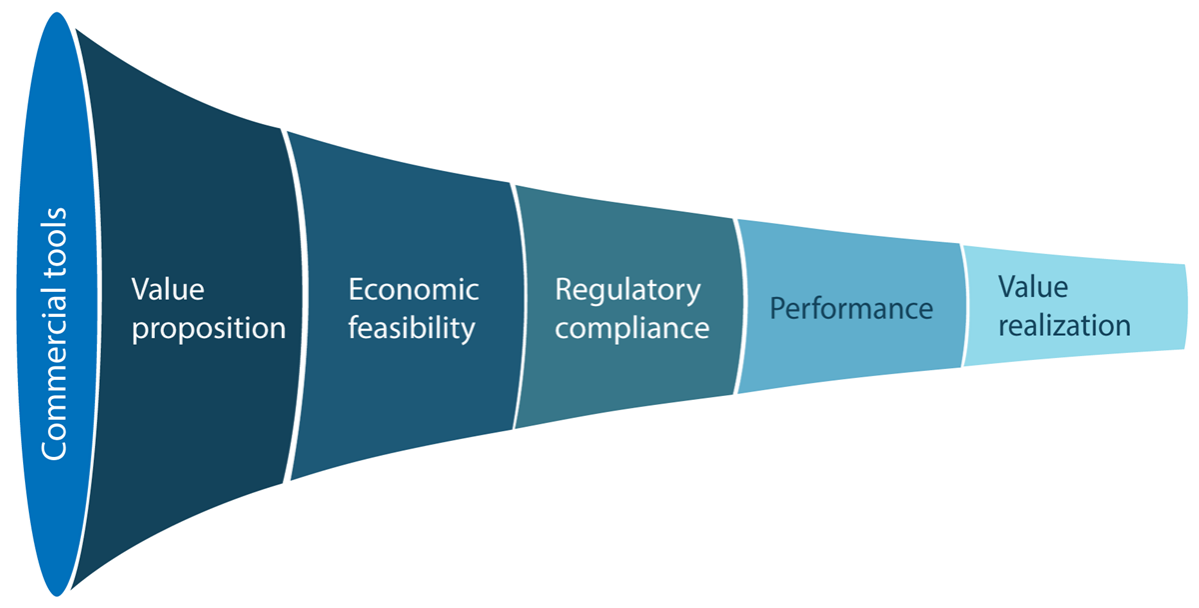
Technology selection of artificial intelligence solutions in radiology. Candidate commercial tools are evaluated through sequential criteria, including value proposition, economic feasibility, regulatory compliance, performance, and value realization.

In traditional design thinking, the prototyping phase follows ideation. In the context of health care technology implementation, however, once potential solutions have been identified through technology scouting, this step is operationalized as the triage of commercially available tools (Figure 3 and Table 1). Institutions may alternatively choose to develop proprietary algorithms, an objective that can also benefit from the preceding design thinking steps. However, algorithm development has been extensively addressed elsewhere and is not the primary focus here [38].

The selection process begins with a comprehensive review of available AI products that align with the institution’s intended clinical applications [39]. At this stage, it is essential to critically assess each solution’s value proposition, ensuring that it directly addresses the pain points identified during the need assessment phase. Priority should be given to tools offering specific and measurable benefits rather than to those promising broadly defined improvements. Importantly, the evaluation process should involve professionals who are directly engaged in the specific stage of the radiology workflow targeted by the solution. For example, preimaging tools often affect administrative staff and referring clinicians, solutions at the acquisition stage primarily impact technologists and protocol management, and interpretation-focused tools most directly involve radiologists. Engaging these stakeholders enables a more accurate assessment of the solution’s value proposition by individuals who routinely experience the underlying needs in clinical practice. Moreover, their participation facilitates early identification of stage-specific barriers to adoption, including workflow disruption, resistance to change, and training requirements.

Subsequently, institutions must evaluate the economic sustainability of each solution, considering not only licensing fees but also implementation-related expenses, such as cloud services, local hardware, and system integration, as well as maintenance requirements. At this stage, the objective is not to conduct a formal cost-effectiveness analysis but rather to determine whether the solution is financially compatible with institutional resource constraints. Early screening of financial feasibility functions as a gatekeeping step, preventing unnecessary progression to resource-intensive activities, such as technical evaluation and pilot testing, for economically impractical solutions.

Beyond economic feasibility, regulatory status represents a second fundamental eligibility filter in the selection process. Regulatory compliance requirements may vary according to the stage of the radiology workflow targeted by the solution. While applications focused on image acquisition or workflow optimizations often face less stringent regulatory oversight, AI tools with direct clinical applications, such as CDS systems and image interpretation algorithms, require more rigorous evaluation [40], as they are typically classified as software as a medical device (SaMD). International initiatives, such as those led by the International Medical Device Regulators Forum, have helped shape the frameworks guiding SaMD regulation [41]. These principles have been incorporated into the legislation of the European Union [42], the United States [43], and Brazil [44,45]. However, each country maintains its own independent approval process. Therefore, institutions must ensure that the selected solutions hold current clearance from the appropriate regulatory agency in the jurisdiction where implementation is intended.

Once solutions have met relevance, economic feasibility, and regulatory eligibility criteria, performance evaluation becomes the primary basis for comparative decision-making. The performance of each solution must be rigorously evaluated using technical and real-world impact indicators, according to the solution evaluated. For image interpretation applications, technical metrics remain essential and include accuracy, sensitivity, specificity, and predictive values. Importantly, models trained on limited or homogeneous datasets often exhibit reduced generalizability, underperforming when deployed in clinical environments that differ from their development context [46]. Therefore, preference should be given to algorithms validated through external multicenter studies, as these are more likely to demonstrate consistent performance across diverse settings and patient populations [47].

However, even highly accurate models may fail to deliver meaningful clinical value or may encounter resistance to adoption. Accordingly, nontechnical KPIs should also be incorporated into decision-making. For clinical applications, these may include report turnaround time, clinician time saved, alert fatigue, and radiologist satisfaction, whereas for other stages of the radiology workflow, relevant KPIs may include scheduling efficiency, protocol adherence, scan time reduction, or user adoption rates.

Together, these technical and operational performance indicators allow institutions to quantify the real-world impact of the solution’s value proposition. When baseline indicators related to the targeted clinical or operational problem are available, institutions can estimate context-specific effects of implementation. At this point, performance data are used to estimate value realization, whereby quantified impact provides the empirical basis for return-on-investment calculations and more refined cost-effectiveness considerations [39].

Importantly, although economic evaluations are typically grounded in currently available performance evidence, the dynamic nature of AI systems introduces an additional temporal dimension of value, as such tools may undergo iterative improvement. Moreover, early implementation can generate cumulative institutional benefits, including workforce familiarization, workflow optimization, and progressive digital infrastructure development. As a result, the overall value of these solutions may extend beyond their initially observed impact, supporting the incorporation of longitudinal and strategic considerations alongside conventional cost-effectiveness analyses.

### Technical Evaluation

**Figure 4.**
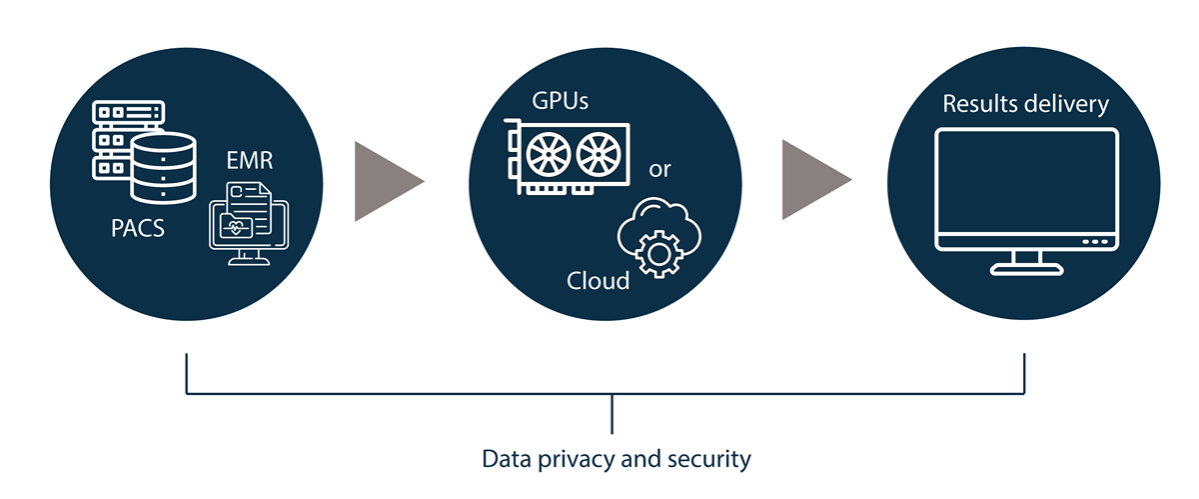
Technical evaluation of artificial intelligence solutions in radiology. Candidate tools are assessed in terms of system architecture (data inputs, processing environment, and delivery strategy) and compliance with data privacy and security requirements. EMR: electronic medical record; PACS: picture archiving and communication system.

After potential solutions have been short-listed based on the criteria discussed above, a rigorous technical evaluation must be conducted by a multidisciplinary IT team (Table 1). This team should ideally include professionals with expertise in systems architecture, data governance, privacy compliance, and information security. The objective of this assessment is to verify that the selected tools can be effectively integrated into the hospital’s existing digital infrastructure and to ensure adherence to established best practices in data protection and cybersecurity, thereby safeguarding both patient information and institutional integrity (Figure 4).

The solution architecture is initially mapped to ensure effective data management and interoperability with institutional systems [8]. This process involves identifying the data inputs and their sources, the processing environment, and the strategy for delivering results to end users. Inputs typically include medical images and associated metadata retrieved from picture archiving and communication systems (PACS) using the standardized Digital Imaging and Communications in Medicine (DICOM) format. Some solutions may also leverage structured clinical information extracted from EMRs, such as patient demographics, comorbidities, and laboratory results. For these integrations, interoperability frameworks developed by Health Level Seven (HL7) International, particularly the Fast Healthcare Interoperability Resources (FHIR) standard, have gained increasing adoption [48]. Processing may occur locally, on dedicated GPUs, or remotely, via cloud-based services. Local processing offers greater control over sensitive health information but demands substantial infrastructure and specialized technical personnel. In contrast, cloud-based solutions offer greater scalability and easier maintenance, though they require reliable connectivity and robust security protocols [49]. A detailed overview of hardware requirements for local GPU configurations has been discussed elsewhere [50].

Additionally, a thorough assessment of data privacy and security must be conducted. Systems must comply with national data protection regulations, particularly regarding principles such as purpose limitation, transparency, and data minimization. Access to sensitive data should be restricted to authorized personnel by robust authentication mechanisms [7]. Encryption and anonymization provide additional layers of protection and should be implemented whenever feasible, either by rendering data unintelligible to unauthorized parties or by fully removing personally identifiable elements. Communication protocols must follow secure data transmission standards, such as HTTPS or SSL/TLS. Finally, disaster recovery plans should be in place to ensure institutional resilience in the event of security breaches, data loss, or system outages.

## Implementation

### Pilot Testing

**Figure 5.**
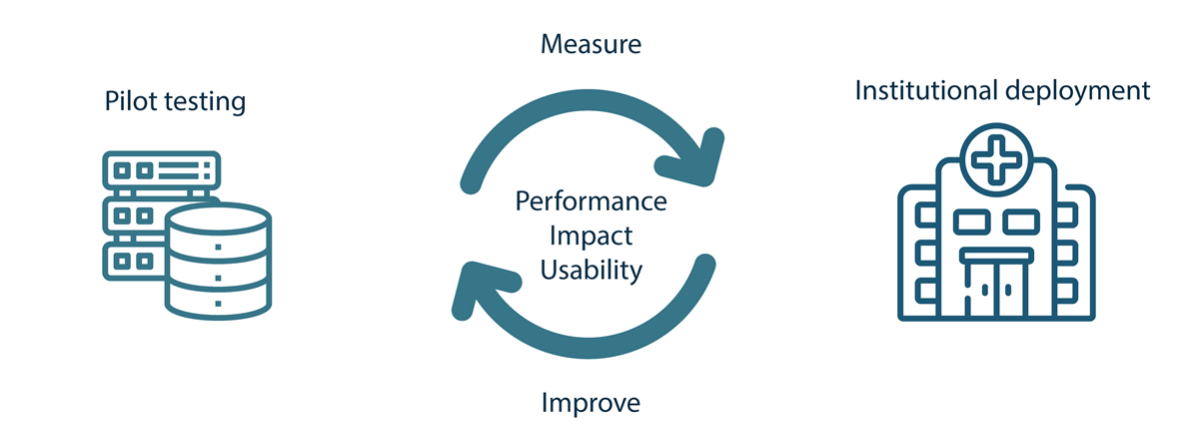
Implementation of artificial intelligence solutions in radiology. Pilot testing is performed in a controlled environment with separated datasets to evaluate algorithm performance, impact, and usability. After institutional deployment, these parameters are continuously monitored to support an iterative improvement cycle.

Selected AI solutions must undergo internal validation in a controlled setting, a process known as pilot testing, to assess their performance, usability, and impact prior to full institutional deployment. This phase serves as a strategic checkpoint to confirm real-world value, identify potential implementation challenges, and build confidence for safe and scalable integration across the organization (Figure 5).

A fundamental objective of pilot testing is to determine whether the AI tool maintains its reported accuracy when applied within the adopting institution’s specific clinical context. As discussed in the technology selection phase, the performance of commercial solutions can be significantly compromised when the algorithm’s training data differs from the local dataset in terms of imaging modalities, workflow characteristics, or patient demographics [6]. Therefore, internal validation using case samples derived from the institution’s own data is essential to reliably assess performance metrics.

Pilot testing also provides a valuable opportunity to assess the broader organizational impact of an AI solution. Measurable clinical and operational benefits should be quantified, as even tools with excellent performance may fail to deliver real-world value depending on the specific context in which they are deployed [51]. For instance, in environments where the baseline workflows are already highly efficient or the volume of applicable cases is low, expected gains in productivity or time savings may prove negligible. While local benefits are often overstated, the resource demands associated with implementation tend to be underestimated. Pilot testing, therefore, enables a realistic appraisal of the solution’s burden on IT infrastructure, including requirements for technical support, system maintenance, and staff training [52]. These data are critical for informing return-on-investment calculations and supporting evidence-based decisions regarding broader institutional scale-up.

The successful adoption of AI tools in radiology also depends heavily on clinician engagement, which is strongly influenced by the perceived usefulness, ease of use, and trust in the system’s output [53]. In this context, pilot testing can be used to assess user experience through systematic collection of structured feedback from radiologists and other end users, with surveys, interviews, or usability testing sessions [9]. This feedback helps identify key barriers to adoption, such as skepticism regarding algorithm reliability, low perceived added value, or disruption to established workflows [52]. When such obstacles are recognized early, targeted strategies can be implemented to enhance acceptance, such as optimizing the user interface or providing tailored training on how to integrate the tool effectively into clinical routines.

To safeguard the interests of all parties involved, this phase should be legally formalized through a well-defined pilot contract that explicitly specifies the scope and objectives of the evaluation, delineates the responsibilities of each party, and incorporates essential clauses addressing compliance, confidentiality, and intellectual property rights. In our institution, we ensure that results are shared with participating companies, fostering collaborative improvements in performance and user experience.

### Feedback-Driven Iteration

Implementing AI in clinical practice is a continuous process of adaptation and refinement. Once a tool is deployed, real-world use often exposes limitations that may not have been fully apparent during pilot testing. Embedding feedback loops into routine operations is, therefore, essential to ensure long-term value and sustained user engagement [54] (Figure 2, bottom).

Iterative improvement depends on the systematic monitoring of both KPIs and user experience [55]. KPIs may include algorithm accuracy, clinical or operational impact, and demands on IT infrastructure. While many of these aspects are initially assessed during pilot testing, the postdeployment phase yields more comprehensive and representative data, owing to the broader scale and greater heterogeneity of use cases. On the user side, continuous education must remain responsive to reported challenges and evolving clinical needs.

Finally, institutions must be prepared to pause or withdraw AI tools that fail to demonstrate sustained value or that impose disproportionate burdens. Thus, feedback-driven iteration is a structural imperative for verifying whether AI technologies deliver their intended value in an effective and sustainable manner.

## Illustrative Example Across Framework Phases

A representative case involved the use of AI to support the interpretation of initial chest radiographs requested for patients presenting with respiratory complaints in the emergency department.

### Opportunity Identification

Workflow mapping and user discussions revealed that, in high-demand clinical settings, frontline clinicians frequently experienced diagnostic uncertainty when interpreting chest radiographs under time pressure. In such contexts, radiologists often prioritized cross-sectional imaging studies (CT and MRI), while lower-complexity examinations such as chest radiographs were reviewed later in the reporting queue. Consequently, initial clinical decisions were frequently made by frontline physicians before radiologist interpretation became available.

The key pain point was, therefore, defined as the lack of immediate decision support at this early stage of care. While clinicians were generally able to recognize the primary abnormality related to the presenting complaint (eg, consolidation in suspected pneumonia), additional or incidental yet clinically relevant findings, such as pulmonary nodules or pneumothorax, were at risk of being overlooked under conditions of high workload and cognitive pressure.

### Solution Selection

A screening of commercially available tools was conducted to identify solutions capable of supporting frontline physicians in detecting high-risk findings without disrupting existing workflow speed. Because these examinations generated limited revenue, economic screening focused on ensuring that implementation costs were compatible with institutional constraints and justified by anticipated safety and efficiency gains. Candidate tools were further assessed for regulatory compliance in accordance with CDS requirements. Performance evaluation included both technical metrics (eg, algorithm accuracy) and real-world indicators, such as reduction in missed critical findings. Although baseline institutional data on missed findings were not available, value realization in this context would rely on estimating the expected reduction in missed cases and translating this effect into institutional impact relative to implementation costs for return-on-investment calculations. The selected tool also underwent technical evaluation to verify compatibility with the institution’s digital infrastructure. The implemented architecture enabled automated retrieval of chest radiographs from PACS for algorithm processing, with AI-generated findings returned as notifications directly within the PACS environment.

### Implementation

The tool was initially deployed in a controlled pilot setting, enabling identification of practical adoption challenges. For example, AI-generated images created confusion among clinicians and radiology technicians who had not received prior training, underscoring the importance of structured onboarding and communication strategies as key determinants of effective adoption.

## Conclusion

Design thinking offers a valuable framework for guiding the implementation of AI technologies in radiology. As discussed in this viewpoint, its user-centered approach facilitates the selection of tools with meaningful impact, tailored to the institution’s specific clinical and operational needs. The emphasis on collaborative decision-making fosters the multidisciplinary coalition necessary to navigate through the technical, ethical, and organizational complexities of AI adoption in health care. Finally, its iterative nature supports continuous refinement based on real-world validation of performance, clinical impact, user experience, and infrastructure demands.

## Abbreviations

AI: artificial intelligence
CDS: clinical decision support
CT: computed tomography
DICOM: Digital Imaging and Communications in Medicine
DL: deep learning
EMR: electronic medical record
FHIR: Fast Healthcare Interoperability Resources
HL7: Health Level Seven
KPI: key performance indicator
MRI: magnetic resonance imaging
PACS: picture archiving and communication system
SaMD: software as a medical device
